# Epidemiology of skateboarding-related injuries sustained by children and adolescents 5-19 years of age and treated in US emergency departments: 1990 through 2008

**DOI:** 10.1186/s40621-016-0075-6

**Published:** 2016-04-08

**Authors:** Lara B. McKenzie, Erica Fletcher, Nicolas G. Nelson, Kristin J. Roberts, Elizabeth G. Klein

**Affiliations:** 1 Center for Injury Research and Policy, The Research Institute at Nationwide Children’s Hospital, 700 Children’s Drive, Columbus, 43205 OH USA; 2 Department of Pediatrics, College of Medicine, The Ohio State University, Columbus, OH USA; 3 Division of Epidemiology, College of Public Health, The Ohio State University, Columbus, OH USA; 4 Division of Health Behavior & Health Promotion, College of Public Health, The Ohio State University, Columbus, OH USA

**Keywords:** Skateboarding, Injury, Children, Adolescents, National electronic injury surveillance system

## Abstract

**Background:**

The goal was to examine the patterns and trends of skateboarding-related injuries sustained by children and adolescents in the United States.

**Methods:**

A retrospective analysis was conducted using data from the National Electronic Injury Surveillance System for children and adolescents 5-19 years of age treated in emergency departments for injuries associated with skateboards from 1990 through 2008.

**Results:**

An estimated 1 226 868 children/adolescents (95 % CI: 948 733—1 505 003) were treated in emergency departments for skateboarding-related injuries from 1990 through 2008, an average of 64,572 cases per year. From 1990 through 1994, the annual rate of injuries per 10,000 children/adolescents significantly decreased overall and for males (overall: 72.9 %, *P* = 0.014; males: 73.9 %, *P* = 0.011; females: 63.6 %, *P* = 0.062). From 1994 to 2008, annual rates of injuries per 10,000 children/adolescents significantly increased overall and for both males and females (overall: 378.9 %, *P* < 0.001; males: 393.4 %, *P* < 0.001; females: 283.3 % *P* < 0.001). From 1990 to 1994 the annual rate of injuries per 10,000 children/adolescents significantly decreased for all age groups (5-10 years: 69.9 %, *P* = 0.043; 11-14 years: 80.6 %, *P* = 0.017; 15-19 years: 64.2 %, *P* = 0.024), and then significantly increased from 1994 to 2008 (5-10 years: 164.5 %, *P* < 0.001; 11-14 years: 587.0 %, *P* < 0.001; 15-19 years: 407.9 %, *P* < 0.001).

Most patients were male (89.0 %), injured at home (37.3 %) or in the street and/or highway (29.3 %), and were not hospitalized (96.9 %). Patients 11-14 years of age constituted 44.9 % of cases. The most commonly injured body regions were the upper (44.1 %) and lower (31.7 %) extremities. Fractures and dislocations were the most common diagnoses (32.1 %). Children/adolescents 11-14 years of age were hospitalized more often than younger or older children/adolescents. Lower extremity injuries increased with age, while face and head or neck injuries decreased with age.

**Conclusions:**

Skateboarding continues to be an important source of injury for children and adolescents. Further research, using more rigorous study designs, is required develop a broad perspective of the incidence and determinants of injury, and to further identify risk factors and viable injury countermeasures while simultaneously promoting participation in skateboarding.

## Background

Skateboarding has evolved from sidewalk-surfing, on primitive skateboards which were constructed from planks of wood with metal roller skate wheels (Hawkins and Lyne 1981), into a dynamic, adrenaline-filled sport with advanced gear, ramps, skate parks, and extreme-type competitions (Brooke 1999). Skateboarding is a popular recreational sport and participation has increased the last several decades, faster than any other sport or recreation activity between 1998 and 2007 (National Sporting Goods Association 2008). Previous reports have estimated ~50 000 emergency department (ED) visits and 1500 hospitalizations among children and adolescents annually in the United States (US) (Committee on Injury and Poison Prevention 2002). The most common skateboarding-related injuries include fractures, (Hawkins and Lyne 1981; Banas et al. 1992; Sheehan et al. 2003; Zalavras et al. 2005) wrist and ankle strains and sprains, (Kyle et al. 2002) and traumatic brain injuries (TBIs) (Schleimer 2002).

Studies on skateboards injuries have focused on a single year of data, (Kyle et al. 2002; Nathanson et al. 2015) or specific types of injuries such as epiphyseal injuries, (Adams 1979) knee injuries, (Shapiro 1994) fractures (Hawkins and Lyne 1981; Banas et al. 1992; Sheehan et al. 2003; Zalavras et al. 2005), minor TBIs (Schleimer 2002), or head injuries (Tominaga et al. 2015). Other research have only considered injuries treated in a single medical facility or trauma center (Sheehan et al. 2003; Hawkins and Lyne 1981; Banas et al. 1992; Adams 1978; Cass and Ross 1990; Christian and Khan 1980; Forsman and Eriksson 2001; Fyfe and Guion 1978; Hassan and Dorani 1999; Illingworth et al. 1978; Kemm 1978; Kirkpatrick 1980; Macdonald et al. 2006; Maitra 1979; McGeehan et al. 2004; Morgan et al. 1980; Rethnam et al. 2008). Previous case series studies (Burt and Overpeck 2001; Finch et al. 1998; Kyle et al. 2002; Osberg et al. 1998; Powell and Tanz 2004; Schieber et al. 1994; Shapiro 1994; Zalavras et al. 2005) have looked at skateboarding injuries treated in EDs, but have only done so for limited time periods (i.e., two years or less) or longer periods of time but the data are now dated. A more recent study by Keays and Dumas (Keays and Dumas 2014) compared cases of longboarding, a sport that uses longer and wider boards than traditional skateboards, and skateboarding-related injuries. These prior studies do not represent the cyclical nature of skateboarding-related injuries. The variation in participation (estimates range from 5.8 million participants <18 years to 74 million participants >7 years) and rates of injury from skateboarding can only be accurately described by examining multiple years of data. Previous research has found that most injuries occur when a skateboarder loses balance leading to a fall (Clark et al. 2011).

To the best of our knowledge, this is the first nationally representative study to examine the epidemiology of skateboarding-related injuries treated in EDs in the US over a 19-year study period from 1990 through 2008, further the current study extends the age groups under examination to include participants up to 19 years of age. The purpose of this current research was to examine injury rates and long-term epidemiologic trends in skateboarding-related injuries by age and sex.

## Methods

### Data source

Data for children and adolescents who were treated in an US ED, from January 1, 1990 through December 31, 2008, were obtained through the National Electronic Injury Surveillance System (NEISS) operated by the US Consumer Product Safety Commission (CPSC). The NEISS provides data on consumer product- and sports-related injuries treated in US EDs. The NEISS receives data from a network of ~100 hospitals, representing a stratified probability sample of 6 100 hospitals with ≥6 beds and a 24-h ED, including urban, suburban, rural, and children’s hospitals (US Consumer Product Safety Commission 2001). Sample weights, based on the inverse probability of selection, were assigned to each case by the CPSC and were used to generate national estimates. At sampled hospitals, ED medical charts are reviewed by professional NEISS coders, and patients’ age and sex, injury diagnosis, body part injured, locale where the injury occurred, product(s) involved, and disposition from the ED, as well as a narrative describing the incident, are recorded. Population estimates from the US Census Bureau (2011) were used to calculate injury rates per 10 000 children/adolescents 5 to 19 years of age.

### Case selection criteria

All NEISS cases for children/adolescents 5 to 19 years of age with a product code of skateboards (code 1333) were reviewed (*n* = 36 862). Inclusion and exclusion criteria and variable categories were developed after a review of a subset of case narratives. All case narratives were reviewed by 1 or more authors to ensure that cases met inclusion criteria (i.e., involved the active use of a skateboard). A total of 6 936 cases were excluded because the narratives revealed that the injury did not occur during active use of a skateboard. In addition, all five fatalities were excluded. All fatalities involved patients who were struck by motor vehicles while skateboarding. The final number of actual cases was 29 921.

### Variables

Data regarding patient sex, age, injury diagnosis, body part injured, locale, and disposition were coded as categorical variables. Patients were categorized according to age (5-10, 11-14, and 15-19 years of age). Locale (location where the injury occurred) was categorized as home (including home, apartment, mobile home, and farm), street and/or highway, sports and/or recreation place, school and other (including other public property and industrial space). The body parts injured were grouped according to region, in categories of head (including head and neck), face (including eyes, ears, mouth, and face), upper extremity (including shoulder, upper arm, lower arm, elbow, wrist, hand, and finger), lower extremity (including upper leg, knee, lower leg, ankle, foot and toe), trunk (including upper trunk, lower trunk and pubic region), and other (including “25 %-50 % of the body” and “all parts of body”). Diagnosis was grouped according to injury type, in categories of soft tissue injuries (including contusions, abrasions, and hematomas), lacerations (including lacerations, punctures, and avulsions), fractures and dislocations, internal injury, sprains and strains, traumatic brain injuries (TBIs) (including concussions, fractures of the head, and internal organ injuries of the head), (Xiang et al. 2007) and other (including crushing, foreign body, dental injury, nerve damage, amputation, burns, hemorrhage, and other/not documented). Disposition was categorized as hospitalized (including treated and transferred to another hospital, treated and admitted for hospitalization, or held for observation <24 h) or not hospitalized (including treated and released or examined and released without treatment).

### Statistical analyses

All statistical testing and variance estimation accounted for the complex sampling frame of the NEISS using SURVEY procedures in SAS version 9.3 (SAS Institute, Inc, Cary, NC). Bivariate comparisons were conducted by using Rao-Scott design-corrected chi-square likelihood-ratio *χ*^2^. Linear regression was used to determine change in the rate of skateboarding-related injuries over the study period. Logistic regression was used to identify characteristics of skateboarding-related injuries associated with age. Statistical significance was assessed at α = 0.05 from the Wald *χ*^2^ with the magnitude of association evaluated using odds ratios (ORs) and 95 % confidence intervals (CIs) from univariate logistic regression. All data reported in this article are national estimates unless specified as unweighted cases. The Nationwide Children’s Hospital institutional review board approved this study (approval number 0403HSX066).

## Results

### Overall injury trends

From 1990 through 2008, an estimated 1 226 868 children/adolescents (95 % CI: 948 733—1 505 003) 5-19 years of age were treated in EDs for skateboarding-related injuries (Table 1), an average of 64,572 cases per year. The mean age (standard deviation) was 12.9 (3.24) years, and patients 11-14 years constituted 44.9 % of cases. Most were males (89.0 %), injured at home (37.3 %) or in the street and/or highway (29.3 %), and were not hospitalized (96.9 %). The most commonly injured body regions were the upper (44.1 %) and lower (31.7 %) extremities, followed by the face (11.4 %) and head and/or neck (7.8 %). Fractures and dislocations were the most common diagnoses (32.1 %), followed by sprains and strains (24.8 %), soft tissue injuries (20.0 %), and lacerations (14.3 %).

**Table 1:** Characteristics of skateboarding-related injuries treated in US EDs, 1990-2008

Characteristic	Actual cases (n)^a^	National estimate^a^	% (95 %) CI^b,c,d^
Age, (years)	29 921	1 226 868	100.0
5-10	6471	241 506	19.7 (18.3-21.1)
11-14	13 825	550 694	44.9 (43.6-46.2)
15-19	9625	434 668	35.4 (33.7-37.2)
Sex	29 916	1 226 727	100.0
Boys	26 578	1 092 348	89.0 (88.4-89.7)
Girls	3338	134 379	11.0 (10.3-11.6)
Locale	17 473	752 743	100.0
Home	6527	280 793	37.3 (30.4-44.2)
Street/highway	5281	220 925	29.3 (23.1-35.6)
Sports/recreation place	4120	184 251	24.5 (19.4-29.6)
School	331	15 858	2.1 (1.4-2.8)
Other	1214	50 916	6.8 (5.1-8.4)
Disposition	29 893	1 225 612	100.0
Not hospitalized	28 674	1 187 617	96.9 (96.5-97.3)
Hospitalized	1219	37 995	3.1 (2.7-3.5)
Body part injured	29 868	1 224 461	100.0
Upper extremity^e^	13 216	540 473	44.1 (43.0-45.2)
Lower extremity^f^	9135	388 705	31.7 (30.4-33.1)
Face^g^	3361	139 439	11.4 (10.6-12.1)
Head or neck	2685	95 740	7.8 (7.1-8.5)
Trunk	1395	56 161	4.6 (4.3-4.9)
Other	76	3943	0.3 (0.2-0.4)
Diagnosis	29 884	1 225 631	100.0
Fracture or dislocation	10 052	392 923	32.1 (30.1-34.0)
Sprain or strain	6818	303 499	24.8 (22.9-26.6)
Soft Tissue	5750	245 227	20.0 (18.8-21.2)
Laceration	4059	175 523	14.3 (13.6-15.1)
TBI	1782	56 477	4.6 (3.9-5.3)
Other	1423	51 982	4.2 (3.3-5.2)

^a^Some *n* values may differ due to missing data

^b^Percentages may not total to 100.0 % due to rounding

^c^95 % CIs constructed using sample weights based on the inverse probability of selection in SAS SURVEY procedures

^d^All *P*-values <0.0001 from the Rao-Scott design-corrected chi-square testing proportionality of groups

^e^Upper extremity included finger, hand, wrist, lower arm, elbow, and upper arm

^f^Lower extremity included toe, foot, ankle, lower leg, knee, and upper leg

^g^Face included face, mouth, eyeball, and ears

**Fig. 1:**
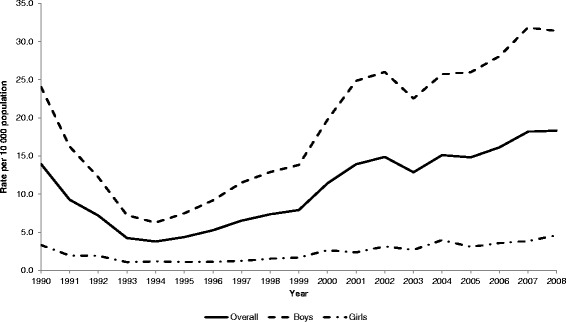
Rates of skateboarding-related injury per 10,000 population estimate overall and by sex, 1990-2008

From 1990 through 1994 the annual rate of injuries per 10,000 children/adolescents significantly decreased overall and for males, while the decrease in injury to females was not significant (overall: 72.9 %, *P* = 0.014; males: 73.9 %, *P* = 0.011; females: 63.6 %, *P* = 0.062). From 1994 to 2008 annual rates of injuries per 10,000 children/adolescents significantly increased overall and for both males and females (overall: 378.9 %, *P* < 0.001; males: 393.4 %, *P* < 0.001; females: 283.3 % *P* < 0.001 (Fig. 1).

### Injury trends by age

**Fig. 2:**
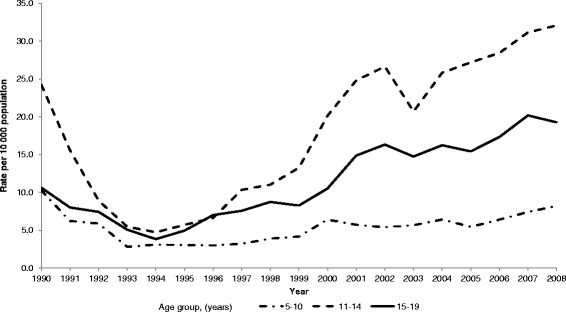
Rates of skateboarding-related injury per 10,000 population estimate by age group, 1990-2008

From 1990 to 1994 the annual rate of injuries per 10,000 children/adolescents significantly decreased for all age groups (5-10 years: 69.9 %, *P* = 0.043; 11-14 years: 80.6 %, *P* = 0.017; 15-19 years: 64.2 %, *P* = 0.024), and then significantly increased from 1994 to 2008 (5-10 years: 164.5 %, *P* < 0.001; 11-14 years: 587.0 %, *P* < 0.001; 15-19 years: 407.9 %, *P* < 0.001) (Fig. 2).

Children/adolescents 5-10 years of age had the highest proportion of females (18.4 %), were most commonly injured at home (51.9 %) or in the street and/or highway (24.2 %), and had the lowest proportion of hospitalizations (2.6 %) (Table 2). These children/adolescents most commonly injured the upper extremities (42.3 %), face (26.0 %), and lower extremities (18.1 %). The most common diagnoses for children/adolescents 5-10 years of age were lacerations (27.6 %), fractures or dislocations (27.0 %) and soft tissue injuries (23.4 %).

**Table 2:** Characteristics of skateboarding-related injuries treated in US EDs by age group, 1990-2008

	Age group					
	5-10 y		11-14 y		15-19 y	
Characteristic	National estimate^a^	% (95 % CI)^b,c^	National estimate^a^	% (95 % CI)^b,c^	National estimate^a^	% (95 % CI)^b,c^
Sex	241 491	100.0	550 575	100.0	434 661	100.0
Boys	196 994	81.6 (80.2-83.0)	491 940	89.3 (88.5-90.2)	403 414	92.8 (92.1-93.6)
Girls	44 496	18.4 (170-19.8)	58 635	10.6 (9.8-11.5)	31 247	7.2 (6.4-7.9)
Locale	147 118	100.0	335 768	100.0	269 856	100.0
Home	76 419	51.9 (43.8-60.1)	130 372	38.8 (31.1-46.5)	74 002	27.4 (21.8-33.0)
Street/highway	35 574	24.2 (18.7-29.7)	96 619	28.8 (22.2-35.3)	88 732	32.9 (25.8-39.9)
Sports/recreation place	27 166	18.5 (13.4-23.5)	80 829	24.1 (19.1-29.1)	76 256	28.3 (22.5-34.0)
School	1398	1.0 (0.6-1.3)	5661	1.7 (1.3-2.1)	8799	3.3 (1.6-4.9)
Other	6561	4.5 (3.0-5.9)	22 287	6.6 (4.9-8.3)	22 068	8.2 (6.0-10.3)
Disposition	241 342	100.0	550 177	100.0	434 092	100.0
Not hospitalized	235 166	97.4 (97.0-97.9)	530 385	96.4 (95.9-96.9)	422 065	97.2 (96.8-97.7)
Hospitalized	6176	2.6 (2.1-3.0)	19 792	3.6 (3.1-4.1)	12 027	2.8 (2.3-3.2)
Body Part injured	241 318	100.0	549 391	100.0	433 751	100.0
Upper extremity^d^	102 148	42.3 (40.5-44.2)	272 044	49.5 (48.2-50.8)	166 280	38.3 (36.7-40.0)
Lower extremity^e^	43 622	18.1 (16.8-19.3)	162 688	29.6 (28.1-31.0)	182 414	42.1 (39.8-44.3)
Face^f^	62 806	26.0 (24.1-28.0)	45 133	8.2 (7.6-8.8)	31 520	7.3 (6.5-8.1)
Head or neck	23 814	9.9 (8.8-10.9)	40 832	7.4 (6.7-8.1)	31 094	7.2 (6.3-8.0)
Trunk	8326	3.5 (2.7-4.2)	27 403	5.0 (4.6-5.4)	20 432	4.7 (4.2-5.2)
Other	602	0.2 (0.1-0.4)	1332	0.2 (0.1-0.4)	2009	0.5 (0.3-0.7)
Diagnosis	241 073	100.0	550 346	100.0	434 212	100.0
Fracture or dislocation	64 995	27.0 (24.5-29.4)	200 933	36.5 (34.5-38.5)	126 995	29.2 (27.1-31.4)
Sprain or strain	28 025	11.6 (10.4-12.8)	128 665	23.4 (21.5-25.2)	146 808	33.8 (31.1-36.6)
Soft tissue	56 348	23.4 (21.4-2.3)	111 613	20.3 (18.9-21.7)	77 266	17.8 (16.4-19.2)
Laceration	66 582	27.6 (25.7-29.4)	61 660	11.2 (10.5-11.9)	47 280	10.9 (10.2-11.6)
TBI	12 239	5.1 (4.1-6.0)	25 219	4.6 (3.9-5.2)	19 019	4.4 (3.5-5.3)
Other	12 883	5.3 (4.4-6.3)	22 255	4.0 (3.0-5.1)	16 843	3.9 (2.8-5.0)

^a^Some *n* values may differ due to missing data

^b^Percentages may not total to 100.0 % due to rounding

^c^95 % CIs constructed using sample weights based on the inverse probability of selection in SAS SURVEY procedures

^d^Upper extremity included finger, hand, wrist, lower arm, elbow, and upper arm

^e^Lower extremity included toe, foot, ankle, lower leg, knee, and upper leg

^f^Face included face, mouth, eyeball, and ears

Children/adolescents 11-14 years of age were most commonly injured at home (38.8 %) or the street and/or highway (28.8 %), and had the highest proportion of hospitalizations (3.6 %) (Table 2). Children/adolescents 11-14 years of age most commonly injured the upper (49.5 %) or lower extremities (29.6 %) and the most common diagnoses were fractures or dislocations (36.5 %), sprains or strains (23.4 %), and soft tissue injuries (20.3 %).

Children/adolescents 15-19 years of age had the lowest proportion of females (7.2 %), the highest proportion of males (92.8 %), and were most commonly injured in the street and/or highway (32.9 %) or a sports and/or recreation place (28.3 %) (Table 2). Children/adolescents 15-19 years of age most commonly injured the lower (42.1 %) or upper extremities (38.3 %), and the most common diagnoses were sprains or strains (33.8 %) and fractures or dislocations (38.3 %).

The odds of injury for each characteristic, across age groups, are shown in Table 3. For females, as age increased, the proportion of injuries sustained at home decreased, while injuries sustained in the street and/or highway or sports and/or recreation place increased. Children/adolescents 11-14 years of age had greater odds of hospitalization compared to younger children (OR: 1.42 (1.17-1.73)), while older and younger children/adolescents had similar odds of hospitalization (OR: 1.09 (0.89-1.33)). Lower extremity injuries increased with age, while face and head or neck injuries decreased with age. TBIs were higher for children/adolescents 5-10 years of age than older children/adolescents, but this difference was not statistically significant (OR: 1.17 (0.96-1.42)).

**Table 3:** Differences in characteristics of skateboarding-related injuries treated in US EDs across age groups, 1990-2008

	Age group OR (95 % CI)^a^		
Characteristic	5-10 y	11-14 y	15-19 y
Sex			
Girls	**2.92 (2.54-3.35)**	**1.54 (1.37-1.73)**	*1 (referent)*
Locale			
Home	**2.86 (2.42-3.38)**	**1.68 (1.49-1.89)**	*1 (referent)*
Street/highway	*1 (referent)*	**1.27 (1.06-1.51)**	**1.54 (1.23-1.91)**
Sports/recreation place	*1 (referent)*	**1.40 (1.22-1.61)**	**1.74 (1.46-2.07)**
School	*1 (referent)*	**1.79 (1.22-2.63)**	**3.51 (1.93-6.38)**
Disposition			
Hospitalized	*1 (referent)*	**1.42 (1.17-1.73)**	1.09 (0.89-1.33)
Body part injured			
Upper extremity^b^	**1.18 (1.07-1.30)**	**1.58 (1.48-1.69)**	*1 (referent)*
Lower extremity^c^	*1 (referent)*	**1.91 (1.74-2.09)**	**3.29 (2.91-3.71)**
Face^d^	**4.49 (3.86-5.23)**	1.14 (1.00-1.31)	*1 (referent)*
Head or neck	**1.42 (1.25-1.61)**	1.04 (0.93-1.16)	*1 (referent)*
Trunk	*1 (referent)*	**1.47 (1.17-1.85)**	**1.38 (1.10-1.74)**
Diagnosis			
Fracture or dislocation	*1 (referent)*	**1.56 (1.42-1.72)**	**1.12 (1.01-1.24)**
Sprain or strain	*1 (referent)*	**2.32 (2.08-2.59)**	**3.88 (3.41-4.43)**
Soft tissue injury	**1.41 (1.27-1.57)**	**1.18 (1.06-1.30)**	*1 (referent)*
Laceration	**3.12 (2.78-3.51)**	1.03 (0.93-1.14)	*1 (referent)*
TBI	1.17 (0.96-1.42)	1.05 (0.90-1.22)	*1 (referent)*

^a^ORs and 95 % CIs from univariate logistic regression models, statistically significant ORs are bold and reference age group is labeled as ‘*1 (referent)’*

^b^Upper extremity included finger, hand, wrist, lower arm, elbow, and upper arm

^c^Lower extremity included toe, foot, ankle, lower leg, knee, and upper leg

^d^Face included face, mouth, eyeball, and ears

## Discussion

During the 19-year study period, more than 1.2 million skateboarding-related injuries were treated in US EDs. Population-based rates of skateboarding-related injuries among children and adolescents 5-19 years of age have increased since 1994 for both males and females and among all age groups. Similar to previously published studies, the majority of skateboarding-related injuries occurred to males. Children and adolescents 11-14 years of age were the age group most affected, comprising almost one-half of the total injuries (44.9 %), which may reflect an increase in participation in skateboarding and/or a lower skill level than older children/adolescents. Despite the lack of specific injury severity data in the NEISS dataset, using hospitalization as a proxy for severity, the vast majority of skateboarding-related injuries in the current study were not severe as they were able to be treated in the ED and not hospitalized (96.9 %). Similar to previous research that found that upper extremity and lower extremity injuries were the most common injury (Clark et al. 2011), most injuries in this study were to the upper extremities (44.1 %) and many were fractures and dislocations (32.1 %). A study published in 2002 also utilizing the NEISS data found the most frequent injuries were ankle strains/sprains and wrist fractures; injury rates were twice as high as in-line skating and one-half as high as basketball-related injuries during the same period (Kyle et al. 2002).

There were also notable differences in the type of injuries when compared across age groups, body parts injured and diagnoses. Younger children/adolescents 5-10 years of age sustained more injuries to the face. Children/adolescents 11-14 years of age sustained the most upper extremity injuries; and 15-19 year olds sustained the most lower extremity injuries. Overall, most skateboarding-related injuries occurred at home (37.3 %) or in the street and/or highway (29.3 %), places that were likely easily accessible. Older children/adolescents were more likely to be injured at school, street and/or highway, and at sports and/or recreational facilities. If they were injured in the street, they were more likely to be hospitalized. Females were more likely to be injured at home.

Recommendations exist to reduce and prevent injuries associated with skateboarding. The extent to which these recommendations are followed is, unfortunately, unable to be determined with the NEISS data. It is worth noting that current recommendations suggest that children/adolescents <5 years of age should not ride skateboards and children/adolescents between 6 and 10 years of age be closely supervised while skateboarding (Committee on Injury and Poison Prevention 2002). Many skateboarding-related injuries may be able to be prevented if protective gear such as helmets, wrist guards, elbow pads and knee pads are worn. Helmets that comply with the CPSC-standard or a multi-sport helmet that complies with the N-94 standard should be worn while skateboarding (Consumer Product Safety Commission 1998). We found that one-third of injuries to adolescents 15-19 years of age, and one-quarter of injuries to younger children/adolescents occurred in the street and/or highway, therefore it is recommended that skateboarding parks (in particular ones that mandate protective equipment use) be developed. Further, skateboarders should not ride in traffic. The fatalities (although not included in these analyses) involved children/adolescents who were struck by motor vehicles. There may have been other fatalities that occurred at the scene and would not be counted in the NEISS. In the current analyses skateboarding-related fatalities were excluded, however, this may be worthy of exploration in future work, as each fatality in the data set was related to a collision with a motor vehicle and injuries that occurred in the street or highway were associated with hospitalization.

### Limitations

This study has several limitations. This study underestimates the total number of injuries because the sample includes only injuries treated in US EDs. Case narratives often did not include whether protective equipment such as helmets, elbow pads, or wrist guards were being worn at the time of the injury. Despite these limitations, the strengths of this study are its large, nationally representative sample and its 19-year study period.

## Conclusions

Skateboarding-related injuries continue to be an important source of injury for children and adolescents. Population-based rates of skateboarding-related injuries among children and adolescents age 5-19 in the US have been increasing since 1994. Further research using more rigorous study designs is required to develop a broad perspective of the incidence and determinants of injury, and to further identify risk factors and viable injury countermeasures while simultaneously promoting participation in skateboarding.

## Abbreviations

CI: confidence interval
CPSC: consumer product safety commission
ED: emergency department
NEISS: National Electronic Injury Surveillance System
TBI: traumatic brain injury
US: United States
